# Hydrogen Sulfide From Cysteine Desulfurase, Not 3-Mercaptopyruvate Sulfurtransferase, Contributes to Sustaining Cell Growth and Bioenergetics in *E. coli* Under Anaerobic Conditions

**DOI:** 10.3389/fmicb.2019.02357

**Published:** 2019-10-11

**Authors:** Jun Wang, Xin Guo, Heng Li, Haizhen Qi, Jing Qian, Shasha Yan, Junling Shi, Weining Niu

**Affiliations:** School of Life Sciences, Northwestern Polytechnical University, Xi’an, China

**Keywords:** hydrogen sulfide, cysteine desulfurase, bioenergetics, anaerobic conditions, 3-mercaptopyruvate sulfurtransferase

## Abstract

Endogenous hydrogen sulfide (H_2_S), which is primarily generated by 3-mercaptopyruvate sulfurtransferase (3-MST) in *Escherichia coli* (*E. coli*) under aerobic conditions, renders bacteria highly resistant to oxidative stress. However, the biosynthetic pathway and physiological role of this gas under anaerobic conditions remains largely unknown. In the present study, we demonstrate that cysteine desulfurase (IscS), not 3-MST, is the primary source of endogenous H_2_S in *E. coli* under anaerobic conditions. A significant decrease in H_2_S production under anaerobic conditions was observed in *E. coli* upon deletion of IscS, but not in 3-MST-deficient bacteria (Δ*mstA*). Furthermore, the H_2_S-producing activity of recombinant IscS using *L*-cysteine as a substrate exhibited an approximately 2.6-fold increase in the presence of dithiothreitol (DTT), indicating that H_2_S production catalyzed by IscS was greatly increased under reducing conditions. The activity of IscS was regulated under the different redox conditions and the midpoint redox potential was determined to be −329 ± 1.6 mV. Moreover, in *E. coli* cells H_2_S production from IscS is regulated under oxidative and reductive stress. A mutant *E. coli* (Δ*iscS*) strain lacking a chromosomal copy of the IscS-encoding gene *iscS* showed significant growth defects and low levels of ATP under both aerobic and anaerobic conditions. The growth defects could be fully restored after addition of 500 μM Na_2_S (an H_2_S donor) under anaerobic conditions, but not by the addition of cysteine, sodium sulfite or sodium sulfate. We also showed that the addition of 500 μM Na_2_S to culture medium stimulates ATP synthesis in the mutant *E. coli* (Δ*iscS*) strain in the logarithmic growth phase but suppresses ATP synthesis in wild-type *E. coli*. Our results reveal a new H_2_S-producing pathway in *E. coli* under anaerobic conditions and show that hydrogen sulfide from IscS contributes to sustaining cell growth and bioenergetics under oxygen-deficient conditions.

## Introduction

The noxious gas hydrogen sulfide (H_2_S) is now recognized as a third gas signaling molecule together with nitric oxide (NO) and carbon monoxide (CO), which play important roles in mammals, including in inflammation, vascular tone, angiogenesis, cancer, and protection against oxidative stress (Chen et al., 2012; Kimura, 2014; Niu et al., 2018a). In addition, H_2_S can donate electrons to the mitochondrial electron transport chain through sulfide:quinone oxidoreductase, consequently promoting oxidative phosphorylation and increasing mitochondrial ATP production (Fu et al., 2012; Módis et al., 2016). In mammalian cells, the enzymatic process for endogenous H_2_S production from cysteine is primarily associated with cystathionine β-synthase (CBS, EC 4.2.1.22), cystathionine γ-lyase (CSE, EC 4.4.1.1) and 3-mercaptopyruvate sulfurtransferase (3-MST, EC 2.8.1.2) in combination with cysteine aminotransferase (CAT, EC 2.6.1.3) (Kabil and Banerjee, 2014; Niu et al., 2018b).

Compared with H_2_S production derived from mammalian cells, the bacterial production of H_2_S was described in the 19^*th*^ century but was considered to be a byproduct of sulfur metabolism that lacked physiological roles (Shatalin et al., 2011; Kimura, 2014). Consequently, few studies have been published on the H_2_S-associated metabolic pathways in bacteria. Recently, Evgeny Nudler et al. reported that most bacterial genomes, if not all, have orthologs of mammalian CSE, CBS or 3-MST, suggesting that these genes may have had important biological functions throughout bacterial evolution (Shatalin et al., 2011). Indeed, in *Escherichia coli* (*E. coli*) grown in Luria-Bertani (LB) broth, H_2_S is primarily generated by 3-MST under aerobic culture conditions and can defend against oxidative stress induced by antibiotics (Shatalin et al., 2011; Mironov et al., 2017). However, most recently, Sergey Korshunov et al. reported that H_2_S production in 3-MST-deficient *E. coli* (Δ*mstA*) grown in minimal medium was not significantly altered compared with that in wild-type *E. coli* (Korshunov et al., 2016). The basis for the discrepancy between these two results is not known but could result from different components of the culture medium. Since microorganisms that colonize mammalian intestines grow under the oxygen-deficient conditions, the H_2_S biosynthetic pathway in *E. coli* and its physiological functions under anaerobic conditions should be characterized.

Cysteine desulfurase (IscS, EC 2.8.1.7) is a pyridoxal phosphate (PLP)-containing enzyme that catalyzes the conversion of cysteine to alanine and sulfane sulfur via the formation of a protein-bound cysteine persulfide intermediate on a conserved cysteine residue, and this enzyme is highly conserved throughout all kingdoms of life (Zheng et al., 1993; Mihara and Esaki, 2002; Li et al., 2006). Cysteine desulfurase acts as a sulfur donor and is involved in biological sulfur trafficking and the assembly of iron-sulfur clusters, which are essential prosthetic groups required for enzymatic catalysis and respiratory chain complexes (Rouault, 2012). In addition, IscS is capable of donating the persulfide sulfur atoms to a variety of biosynthetic pathways for sulfur-containing biofactors, such as thiamin, transfer RNA thionucleosides, biotin and lipoic acid (Hidese et al., 2011). However, a previous study showed that a cysteine desulfurase from *Azotobacter vinelandii* catalyzes the synthesis of H_2_S and alanine in the presence of dithiothreitol (Zheng et al., 1993). In the *in vitro* studies in which sulfur transfer from IscS to acceptors has been reported, reaction mixtures contained levels of reducing agent (1–5 mM DTT) sufficient to release persulfide-bound sulfur from IscS as H_2_S (Zheng et al., 1993, 1994; Urbina et al., 2001). In plants, cysteine desulfurase is also one of the key enzymes involved in H_2_S biogenesis (Scuffi et al., 2014). Based on these results, we hypothesize that cysteine desulfurase in *E. coli* is probably involved in the synthesis of H_2_S under anaerobic conditions and that endogenous H_2_S production may sustain cellular bioenergetics under oxygen-deficient conditions.

In this study, we report that endogenous H_2_S production in *E. coli* under anaerobic conditions was primarily generated from cysteine desulfurase but not 3-mercaptopyruvate sulfurtransferase. A mutant *E. coli* (Δ*iscS*) strain lacking IscS activity was observed to display a remarkable decrease in H_2_S production under anaerobic conditions, but not under aerobic conditions. H_2_S generated by purified recombinant IscS exhibited an approximately 2.6-fold increase in the presence of DTT. Furthermore, the addition of exogenous Na_2_S (an H_2_S donor), but not cysteine, sodium sulfite or sodium sulfate can stimulate ATP synthesis in the mutant *E. coli* (Δ*iscS*) strain under anaerobic conditions, indicating that H_2_S from cysteine desulfurase in *E. coli* contributes to sustaining cell growth and bioenergetics.

## Materials and Methods

### Strains, Plasmids and Chemicals

The plasmid pET-28a (Novagen, United States) was used for IscS expression. *E. coli* DH5α (Tiangen, China) was used to amplify recombinant plasmids, and *E. coli* BL21(DE3) pLysS (Tiangen) was the host strain for IscS expression. *E. coli* BW25113 and the mutant strain BW25113 (Δ*iscS*) were kindly provided by Jingdan Liang from Shanghai Jiao Tong University (An et al., 2012). The mutant strain BW25113 (Δ*cyuA*) was kindly provided by Sheng Yang from Chinese academy of science. Sodium phenylpyruvate (PPNa) and diamide were obtained from TCI (Shanghai, China). Dithiothreitol (DTT), *trans*-4,5-dihydroxy-1,2-dithiane (DTT_oxi_), 7-Azido-4-methylcoumarin, lead nitrate and *L*-cysteine were obtained from Sigma-Aldrich (St. Louis, United States). The other materials used in this study were purchased from Sangon (Shanghai, China). Unless otherwise specified, all chemicals were used as they were received.

### Expression and Purification of IscS

The *E. coli* cysteine desulfurase *iscS* gene GenBank (Gene ID: 947004) was cloned using *E. coli* DH5α genomic DNA as template for PCR. All the primers used in this study are listed in Supplementary Table S1. To express IscS in *E. coli*, the *iscS* gene was inserted into the *Bam*HI and *Xho*I sites of the expression vector pET-28a in the correct reading frame and was transformed into *E. coli* BL21(DE3) pLysS.

Recombinant *E. coli* cells with the IscS expression constructs were grown in LB medium containing kanamycin (50 μg/ml) and chloramphenicol (50 μg/ml) until the optical density at 600 nm (OD_600_) reached 0.6–0.8. The expression of IscS was induced by adding 0.2 mM isopropyl β-D-1-thiogalactopyranoside (IPTG), and the cells were further cultured for 16 h at 30°C. The harvested cells were resuspended in lysis buffer containing 50 mM phosphate buffered saline (PBS; pH 7.4), 300 mM NaCl and 30 mM imidazole. The resuspended cells were then sonicated using an Ultrasonic Cell Disruption System (Scientz, China). Subsequently, the supernatant was collected by centrifugation at 12,000 rpm for 30 min at 4°C and was loaded onto a HisTrap FF column (GE Healthcare, United States) pre-equilibrated with lysis buffer. The recombinant IscS was eluted with 300 mM imidazole in 50 mM PBS, pH 7.4, and the collected fractions were desalted using a HiTrap Desalting column (GE Healthcare) that had been equilibrated with 50 mM Tris-HCl, pH 8.0. The fractions containing IscS protein were pooled and stored at −80°C. Protein concentration was determined by the BCA protein assay reagent kit (TransGen Biotech, China), and bovine serum albumin was used as a standard.

### Characterization of Purified IscS

The activity of IscS was quantified using the methylene blue method as previously described, with the following modifications (Urbina et al., 2001). First, H_2_S production in the reaction is trapped by Pb(NO_3_)_2_ to produce lead sulfide (PbS). Subsequently, in acid solution PbS reacts with *N, N*-dimethyl-*p*-phenylenediamine to produce methylene blue in the presence of FeCl_3_. The reaction mixture (total volume of 200 μl) contained 50 mM Tris, 1 mM DTT, 0.4 mM [Pb(NO_3_)_2_], pH 8.0, and 5 μg IscS. The reaction was initiated by the addition of 1 mM *L*-cysteine and was incubated for 30 min at 37°C, after which the reaction was terminated by the addition of 25 μl of 20 mM *N*, *N-*dimethyl-*p*-phenylenediamine in 7.2 M HCl and 25 μl of 30 mM FeCl_3_ in 1.2 M HCl. After incubating for 15 min, the samples were centrifuged at 12,000 rpm for 10 min. The supernatants were then transferred to 96-well plates, and the absorbance was determined at 670 nm in a multifunctional microplate reader. The H_2_S concentration was calculated using a standard curve that was prepared using different concentrations of sodium sulfide.

The effect of the redox potential on the activity of IscS was determined in solutions with various redox potentials as previously described (Niu et al., 2018b). Redox buffers were prepared in a 50 mM Hepes buffer containing various concentrations of DTT (9.9–0.025 mM) and DTT_oxi_ (0.1–9.975 mM), pH 7.4. The total DTT concentration in the redox buffers was 10 mM, and all solutions were de-oxygenated by bubbling with nitrogen for 30 min. The DTT/DTT_oxi_ redox potential was calculated according to the Nernst equation (Eq. 1),

$$E_{h}=E_{0}+\frac{R\,T}{n\,F}\times \text{ln}\,\left(\frac{\left[{\text{DTT}}_{\text{oxi}}\right]}{{\left[\text{DTT}\right]}^{2}}\right)\,\left(1\right)$$

Where *E*_0_ = −352 mV at pH 7.4, *R* is the gas constant, *T* is the absolute temperature, and *F* is Faraday’s constant, *n* = 2, and [DTT_oxi_] and [DTT] are molar concentrations of oxidized and reduced DTT, respectively. Subsequently, recombinant IscS (5 μg) was preincubated in solutions with different redox potentials for 1 h at 37°C, and the reaction was initiated by the addition of 10 mM *S*-methylcysteine. After incubation for 30 min at 37°C, the H_2_S-producing activity of IscS was measured using the methylene blue method.

The effect of pH on the activity of IscS was determined using two buffer systems, sodium phosphate (pH 6.0–7.5) and Tris-HCl (pH 8.0–10.5) in the presence of 1 mM DTT. The kinetics of IscS activity was investigated using varying concentrations of *L*-cysteine in the presence 1 mM DTT, and the kinetic constant *K*m was calculated using non-linear regression in GraphPad Prism version 7 (Martin, 1997).

### H_2_S Production Detection

A lead nitrate detection method was used to monitor H_2_S production in the wild-type and mutant *E. coli* cells as previously described (Mustafa et al., 2009). Filter paper (Whatman, United Kingdom) saturated with 2% [Pb(NO_3_)_2_] was affixed to the culture bottle mouth above the level of the liquid culture. Overnight cultures were diluted 1:100 in LB medium and incubated for 8 h at 37°C. To anaerobically culture the wild-type and mutant *E. coli* strains, the culture medium was deoxygenated by nitrogen bubbling for 10 min. Stained paper was scanned and quantified using Image-Pro Plus 6.0, and the results were normalized by OD_600_.

A commercially available fluorescent H_2_S probe 7-azido-4-methylcoumarin (AzMC) was used to measure H_2_S production in cells (Chen et al., 2013). The mutant *E. coli* strains (Δ*mstA* and Δ*iscS*) were cultured in a sealed culture bottle at 37°C under anaerobic conditions. After 3 h of culture, H_2_O_2_ and DTT were injected into the sealed culture bottles at a final concentration of 1 mM and 5 mM respectively followed by incubation for 1 h at 37°C. Subsequently, the cultures were collected by centrifugation at 4,000 rpm at 4°C for 10 min followed by washing 3 times with ice-cold PBS. The precipitate was resuspended in PBS containing 10 μM 7-Azido-4-methylcoumarin (AzMC). The cell suspension was lysed by five cycles of repetitive rapid freezing in liquid nitrogen and thawing in a 37°C water bath. The supernatant was collected by centrifugation at 15,000 rpm at 4°C for 5 min, and the fluorescent intensity was measured with a F-4500 fluorescence spectrophotometer (λex = 365 nm and λem = 450 nm).

### Gene Knockouts of *mstA* and *sufS*

The Δ*mstA*, Δ*iscS*Δ*mstA* and Δ*sufS* mutant derivatives of strain BW25113 were created using the gene knockout method described by Sheng Yang (Jiang et al., 2015), and the plasmid pCas and pTargetF were gifts from Sheng Yang (Addgene plasmid # 62225; Addgene plasmid # 62226) for use in generating the gene knockout strain. All the primers used in this study are listed in Supplementary Table S1. The mutant *E. coli* strains were confirmed by PCR and DNA sequencing.

### Measurement of Growth Curves

Fifty microliters of overnight culture (1%, v/v) was inoculated into a culture bottle containing 5 ml of LB medium with the appropriate chemicals as described in the text or figure legends. For aerobic conditions, *E. coli* was grown at 37°C with shaking (250 rpm). For anaerobic conditions, all the cultures were deoxygenated by nitrogen bubbling for 10 min, and *E. coli* was cultured in a sealed culture bottle at 37°C with shaking (100 rpm). The OD_600_ values were determined using a spectrophotometer at specific times. To determine the effect of Na_2_S on the growth of the wild-type and mutant *E. coli* strains, different concentrations of Na_2_S were injected into the sealed culture bottles.

### Succinate Dehydrogenase (SDH) and Aconitase (ACO) Activity Assays

The activity of succinate dehydrogenase (SDH) in the wild-type and mutant *E. coli* (Δ*iscS*) grown under anaerobic conditions with or without 500 μM Na_2_S was determined using an SDH activity assay kit (Solarbio, Beijing, China). One unit of SDH activity was defined as the amount of enzyme catalyzing the removal of 1 nmol 2,6-dichlorophenol indophenol per minute at 37°C under the specified conditions. The activity of aconitase (ACO) was measured according to the method described by Yarian et al. (2006). One unit of ACO activity was defined as the amount of enzyme catalyzing the generation of 1 nmol NADPH per minute at 37°C.

### Detection of ATP Content in *E. coli* Cells

The ATP content in *E. coli* cells was determined using the luciferin/luciferase reaction with an ATP Assay Kit (Beyotime, China) following the manufacturer’s instructions. The wild-type *E. coli*, mutant *E. coli* (Δ*isc*S) and *E. coli* (Δ*mstA*) strains were growth at 37°C under aerobic or anaerobic conditions as mentioned above. Next, 50 μl of overnight culture was inoculated into a culture bottle containing 5 ml of LB medium. To determine the effect of Na_2_S on the synthesis of ATP in the wild-type and mutant *E. coli* strains, and Na_2_S solution was injected into the sealed culture bottle at a final concentration of 500 μM. After incubation for 2.5, 5, 7.5, and 10 h, the cultures were centrifuged at 4,000 rpm at 4°C for 10 min to collect the precipitate, which was followed by washing 3 times with ice-cold PBS. The precipitate was resuspended in 500 μl of a 50 mM HEPES buffer containing 500 mM NaCl, pH 7.4. The cell suspension was lysed by 5 cycles of a rapid freeze-thaw procedure that involved freezing in liquid nitrogen followed by thawing in a water bath at 37°C. Subsequently, the supernatant was collected by centrifugation at 15,000 rpm at 4°C for 5 min, and 20 μl of the lysate was added to the reaction solution. Luminescence was measured using a bioluminometer, and the results are presented as nmol ATP/mg protein (Zhang et al., 2014).

## Results

### 3-MST Is Not the Primary Source of H_2_S in *E. coli* Under Anaerobic Conditions

**FIGURE 1:**
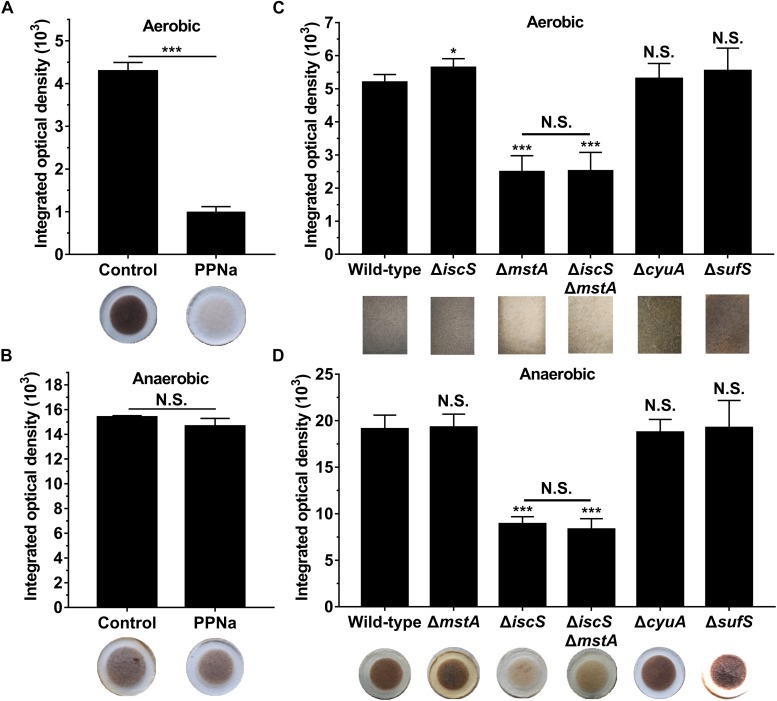
IscS, not 3-MST, is the primary source of H_2_S in *E. coli* under anaerobic conditions. **(A)** H_2_S production in *E. coli* cultured under aerobic conditions. **(B)** H_2_S production in *E. coli* cultured under anaerobic conditions. 3-MST activity was inhibited using 50 mM PPNa. H_2_S production in *E. coli* cultured without PPNa was used as control group. **(C)** H_2_S production in *E. coli* cultures under aerobic conditions. **(D)** H_2_S production in *E. coli* cultures under anaerobic conditions. H_2_S production in *E. coli* cultures was monitored using [Pb(NO_3_)_2_]-soaked paper. Saturated papers were quantified using Image Pro Plus 6.0, and the integrated optical density (IOD) value was normalized to the OD_600_ value of the culture. Each bar represents the mean ± SD of four independent experiments. ^∗^*p* < 0.1, ^∗∗∗^*p* < 0.001, N.S., not significant, versus the control or wild-type group.

To investigate whether 3-MST is the primary source of H_2_S in *E. coli* under anaerobic conditions, PPNa was used to inhibit the activity of 3-MST (Wing and Baskin, 1992). The growth of *E. coli* was not significantly inhibited in the presence of 50 mM PPNa (Supplementary Figure S1). H_2_S production in *E. coli* cultures was monitored using [Pb(NO_3_)_2_]-soaked paper, which specifically reacts with H_2_S to form a brown lead sulfide precipitate, where the H_2_S concentration is directly proportional to the change in the rate at which the paper is stained (Mustafa et al., 2009). Under aerobic conditions, 50 mM PPNa remarkably inhibited the biogenesis of H_2_S by 3-MST in wild-type *E. coli* (Figure 1A), indicating that 3-MST is the primary source of H_2_S in this bacterium. This result is consistent with those reported by Evgeny Nudler (Shatalin et al., 2011; Mironov et al., 2017). Surprisingly, 50 mM PPNa did not significantly affect the H_2_S production in *E. coli* under anaerobic conditions (Figure 1B). These results suggested that 3-MST is not the primary source of H_2_S in *E. coli* under anaerobic conditions.

### IscS Is the Primary Source of H_2_S in *E. coli* Under Anaerobic Conditions

To further verify that 3-MST is not the primary source of H_2_S in *E. coli* under anaerobic conditions, a 3-MST-deficient *E. coli* (Δ*mstA*) strain and a double mutant (Δ*iscS*Δ*mstA*) strain were constructed. These mutant *E. coli* strains were confirmed by agarose gel electrophoresis and sequencing (Supplementary Figure S2a). Additionally, a recent research showed that an iron-sulfur-containing cysteine desulfidase (CyuA) modulates intracellular cysteine concentrations and is the major anaerobic cysteine-catabolizing enzyme in *E. coli* under the conditions tested (Loddeke et al., 2017). To identify the endogenous source of H_2_S under anaerobic conditions, a CyuA-deficient *E. coli* (Δ*cyuA*) strain was constructed. The mutant *E. coli* (Δ*cyuA*) strain was confirmed by agarose gel electrophoresis and sequencing (Supplementary Figure S2b). In addition, SufS is another cysteine desulfurase in *E. coli* and can also abstract sulfur from cysteine, resulting in the production of alanine and persulfide (Kim et al., 2018; Dunkle et al., 2019). We generated an *E. coli* mutant (Δ*sufS*) strain which was verified by agarose gel electrophoresis and sequencing (Supplementary Figure S2c).

First, we investigated the effect of deleting the *mstA*, *iscS*, *cyuA* and *sufS* genes on H_2_S biosynthesis in *E. coli* under both aerobic and anaerobic conditions. Similar to the results obtained for the wild-type *E. coli* cells treated with the 3-MST inhibitor PPNa, endogenous H_2_S production in the *E. coli* (Δ*mstA*) strain and the double mutant (Δ*iscS*Δ*mstA*) strain greatly decreased under aerobic conditions, but not under anaerobic conditions. Conversely, H_2_S biogenesis in the *E. coli* (Δ*iscS*) mutant and (Δ*iscS*Δ*mstA*) mutant dramatically decreased under anaerobic conditions, but not under aerobic conditions (Figures 1C,D). Additionally, the double mutant (Δ*iscS*Δ*mstA*) strain produced the same level of H_2_S as the mutant *E. coli* (Δ*mstA*) under aerobic conditions and the same level of H_2_S as the mutant *E. coli* (Δ*iscS*) under anaerobic conditions. However, the mutant *E. coli* (Δ*cyuA*) strain grown in LB medium generated wild-type levels of H_2_S both aerobically and anaerobically (Figures 1C,D), which is inconsistent with a previous report that mutant *E. coli* (Δ*cyuA*) grown with cysteine generated substantially less H_2_S under anaerobic conditions (Loddeke et al., 2017). The basis for the discrepancy between these two studies could result from different culture medium and culture conditions. There was no significant difference in endogenous H_2_S product between the mutant (*sufS*) strain and a wild type strain both aerobically and anaerobically (Figures 1C,D). Collectively, these results clearly indicated that IscS is the primary source of H_2_S in *E. coli* under anaerobic conditions, at least under the conditions tested.

**FIGURE 2:**
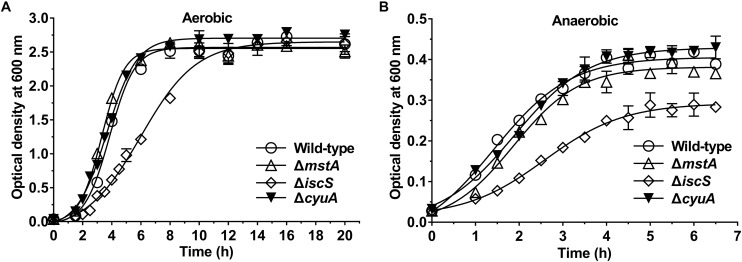
Deletion of the *iscS* gene, but not the *mstA* and Δ*cyuA* gene, significantly decreases the growth rate of *E. coli*. **(A)** Growth curves of the wild-type, Δ*mstA*, Δ*iscS* and Δ*cyuA E. coli* strains under aerobic conditions. **(B)** Growth curves of the wild-type, Δ*mstA*, Δ*iscS* and Δ*cyuA* strains under anaerobic conditions. Each bar represents the mean ± SD of four independent experiments.

We next investigated the effect of the deletion of the *mstA*, *iscS*, *cyuA or sufS* genes on the growth of *E. coli*. As is shown in Figure 2 and Supplementary Figure S3, the deletion of *iscS* significantly decreased the growth rate of *E. coli* under both aerobic and anaerobic conditions, whereas the deletion of *mstA* or *cyuA or sufS* did not.

### The Activity of IscS Is Redox Regulated *in vitro* and in *E. coli* Cell

**FIGURE 3:**
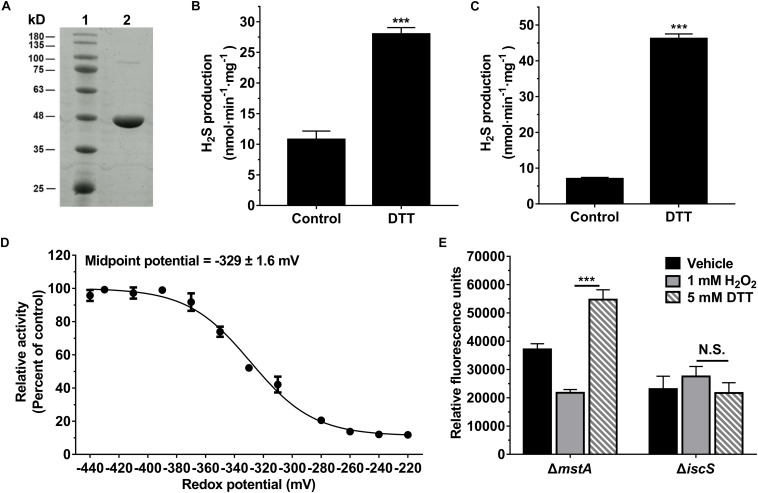
The activity of IscS was redox regulated *in vitro* and in *E. coli* cell. **(A)** SDS-PAGE (12%) analysis of the purified IscS protein: Lane 1 - Molecular mass standards; Lane 2 - Purified IscS protein. **(B)** H_2_S-producing activity of the purified IscS protein using *L*-cysteine as a substrate. Samples without DTT were used as control, and samples without IscS protein were used as blank control, and all values are subtracted by the mean value of blank control. Each bar represents the mean ± SD of four independent experiments. ^∗∗∗^*P* < 0.001, versus the control group. **(C)** H_2_S-producing activity of purified IscS protein using *S*-methylcysteine as a substrate. Samples without DTT were used as control, and samples without IscS protein were used as blank control. ^∗∗∗^*P* < 0.001, versus the control group. **(D)** H_2_S-producing activity of the purified IscS protein was regulated under the different redox conditions. The graph represents the relative activity of the samples compared with control group (samples with 10 mM DTT) and shows the means ± SD (*n* = 4). The H_2_S-producing activity of the control group was 59 nmol⋅min^– 1^⋅mg protein^– 1^. **(E)** H_2_S-producing activity of IscS in *E. coli* cells was redox-regulated under anaerobic conditions. H_2_S production in *E. coli* cell lysates was determined using a fluorescent H_2_S probe AzMC (10 μM). The fluorescent intensity was measured with a fluorescence spectrophotometer (λex = 365 nm and λem = 450 nm). Relative fluorescence units were normalized to the total protein content of each sample. Each bar represents the mean ± SD of four independent experiments.

To assess the H_2_S-producing activity of IscS, the recombinant IscS was purified and the protein purity was judged to be >95% by SDS-PAGE analysis (Figure 3A). The optimum pH of the purified IscS enzyme was 8.0∼8.5 (Supplementary Figure S4a) with a Michaelis constant (*K*m) of 0.8 ± 0.05 mM at pH 8.0 (Supplementary Figure S4b), similar to the characteristics of the human cysteine desulfurase NFS1 (Marelja et al., 2008), but the *K*m value is much higher than that observed for *E. coli* IscS (Urbina et al., 2001). The discrepancy between these two studies could result from different reaction mixtures and reaction conditions. As IscS is a pyridoxal phosphate (PLP)-containing enzyme that generates H_2_S using *L*-cysteine as a substrate, the purified protein appears yellow, which has been observed for other PLP-dependent proteins, and it exhibited a maximum absorbance at 385 nm. When 20 mM cysteine was added to the purified IscS enzyme, the absorbance at 385 nm decreased and a concomitant increase at 350 nm was observed (Supplementary Figure S4c).

The H_2_S-producing activity of the recombinant IscS was assessed in 50 mM Tris-HCl buffer (pH 8.0) using 1 mM *L*-cysteine as a substrate in the absence or presence of DTT. The activity of IscS was determined to be 28 nmol⋅min^–1^⋅mg protein^–1^ in the presence of 1 mM DTT, 2.6-fold higher than that of IscS in the absence of DTT (Figure 3B). IscS can catalyze the formation of a protein-bound persulfide group using *L*-cysteine as a substrate (Lauhon et al., 2004). As *L*-cysteine was used as a substrate and could reduce the persulfide group, a modified assay in which *S*-methylcysteine, a substrate analog of cysteine, served as the substrate was developed. The H_2_S-producing activity of IscS in the presence of DTT (46 nmol⋅min^–1^⋅mg protein^–1^) was 6.3-fold higher than that of IscS in the absence of DTT (Figure 3C). Collectively, these results showed that recombinant IscS has higher specific activity for H_2_S biogenesis under reducing conditions.

We next investigated the effect of the redox potential on the activity of IscS. The activity of IscS in solutions with various redox potentials was determined using the methylene blue method. As shown in Figure 3D, the activity of recombinant IscS for H_2_S generation was regulated under the different redox conditions. The midpoint redox potential was determined to be −329 ± 1.6 mV. Additionally, we determined the H_2_S content of *E. coli* cells using a fluorescent probe under oxidative and reductive stress, which were induced by the addition of 1 mM H_2_O_2_ or 5 mM DTT. As shown in Figure 3E, the H_2_S content of the mutant (Δ*mstA*) strain under reductive stress exhibits a 2.5-fold higher than that under oxidative stress. However, H_2_S production in an IscS-deficient *E. coli* (Δ*iscS*) strain was not affected by the addition of 1 mM or 5 mM DTT. These results indicated that H_2_S production from IscS is redox regulated in *E. coli* cells under anaerobic conditions.

### Exogenous H_2_S Promotes the Growth of the *E. coli* (Δ*iscS*) Mutant Under Anaerobic Conditions

**FIGURE 4:**
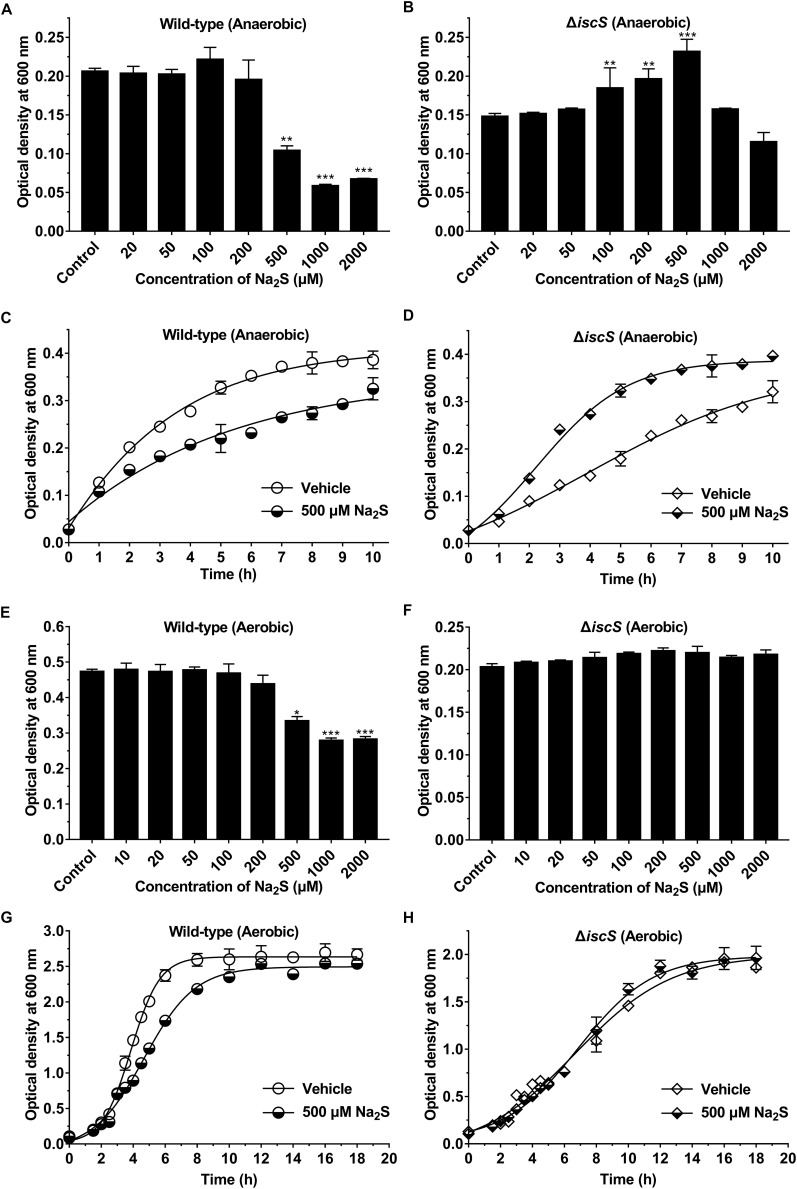
Exogenous H_2_S promotes the growth of the *E. coli* (Δ*iscS*) mutant under anaerobic conditions. **(A)** The effect of different concentrations of Na_2_S on the growth of wild-type *E. coli* under anaerobic conditions. **(B)** The effect of different concentrations of Na_2_S on the growth of the *E. coli* (Δ*iscS*) strain under anaerobic conditions. **(C)** The effect of 500 μM Na_2_S on the growth of wild-type *E. coli* under anaerobic conditions. **(D)** The effect of 500 μM Na_2_S on the growth of the *E. coli* (Δ*iscS*) mutant under anaerobic conditions. **(E)** The effect of different concentrations of Na_2_S on the growth of wild-type *E. coli* under aerobic conditions. **(F)** The effect of different concentrations of Na_2_S on the growth of the *E. coli* (Δ*iscS*) strain under aerobic conditions. **(G)** The effect of 500 μM Na_2_S on the growth of wild-type *E. coli* under aerobic conditions. **(H)** The effect of 500 μM Na_2_S on the growth of the *E. coli* (Δ*iscS*) mutant under aerobic conditions. The data points and errors show the means ± SD of four independent experiments. ^∗^*p* < 0.05, ^∗∗^*p* < 0.01, ^∗∗∗^*p* < 0.001, versus the control group. Samples without Na_2_S were used as a control.

The energy metabolism pathway in *E. coli* is greatly altered under aerobic versus anaerobic conditions. Therefore, we speculated that H_2_S production from IscS would promote the growth of *E. coli* under anaerobic conditions. In the present study, Na_2_S was used as a source of exogenous H_2_S. Indeed, the addition of exogenous Na_2_S significantly increased the intracellular content of H_2_S in *E. coli* cells (Supplementary Figure S5), but did not significantly affect the intracellular content of cysteine (Supplementary Figure S6). First, we investigated the effect of different concentrations of Na_2_S on the growth rates of the wild-type and the mutant *E. coli* (Δ*iscS*) strains under anaerobic conditions. As is shown in Figure 4A, the addition of exogenous Na_2_S at final concentrations of 20–200 μM did not significantly affect the growth of wild-type *E. coli*, whereas 500–2000 μM Na_2_S greatly inhibited its growth (Figures 4A,C). Expectedly, the growth rate of the mutant *E. coli* (Δ*iscS*) strain was significantly promoted by the addition of exogenous Na_2_S at final concentrations of 100–500 μM (Figure 4B), and the cell biomass increased ∼1.6-fold in the presence of 500 μM Na_2_S (Figures 4B,D). Additionally, the effect of Na_2_S on the growth rates of the wild-type and the mutant *E. coli* (Δ*iscS*) strains under aerobic conditions was also determined. As shown in Figure 4E, 500–2000 μM Na_2_S significantly inhibited the growth of wild-type *E. coli* (Figures 4E,G). Interestingly, unlike under anaerobic conditions, 500 μM Na_2_S did not significantly promote the growth of the mutant *E. coli* (Δ*iscS*) strain under aerobic conditions (Figures 4F,H). These results showed that exogenous H_2_S promotes the growth of the *E. coli* (Δ*iscS*) mutant under anaerobic conditions, but not under aerobic conditions.

Additionally, to assess whether Na_2_S specifically promotes the growth of *E. coli*, the effects of *L*-cysteine (0.1–50 mM), sodium sulfite (0.01–5 mM) and sodium sulfate (0.01–5 mM) on the growth of the wild-type and the mutant *E. coli* (Δ*iscS*) strains grown in LB medium and M9 minimal medium under anaerobic conditions were determined. The results indicated that none of the three sulfur compounds could significantly promote the growth of the mutant *E. coli* (Δ*iscS*) (Supplementary Figures S7, S8).

**FIGURE 5:**
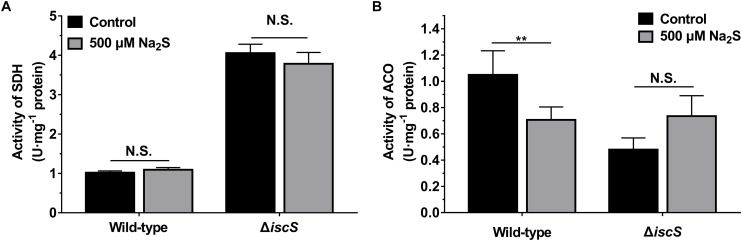
Effect of exogenous H_2_S on the activities of SDH and ACO in *E. coli* cells grown under anaerobic conditions. **(A)** The effect of 500 μM Na_2_S on the activity of SDH. **(B)** the effect of 500 μM Na_2_S on the activity of ACO. Samples without Na_2_S were used as control. Each bar represents the mean ± SD of four independent experiments. ^∗∗^*p* < 0.01, N.S., not significant, versus the control group.

As IscS is involved in the biogenesis of iron-sulfur clusters in *E. coli*, the functions of various Fe-S cluster containing proteins in IscS-deficient mutant *E. coli* (Δ*iscS*) are impaired. To investigate whether exogenous H_2_S rescues Fe-S cluster synthesis in the *E. coli* (Δ*iscS*) mutant, the activities of two Fe-S cluster containing enzymes SDH and ACO in *E. coli* grown with or without 500 μM Na_2_S were determined. As shown in Figure 5, addition of 500 μM Na_2_S to culture medium did not significantly increase the activities of these two enzymes in the mutant *E. coli* (Δ*iscS*) lysate. These results indicated that growth promotion of the mutant *E. coli* (Δ*iscS*) by the addition of Na_2_S should not be attributed to the rescue of Fe-S cluster synthesis.

### Exogenous H_2_S Can Promote ATP Synthesis in the *E. coli* (Δ*iscS*) Strain Under Anaerobic Conditions

**FIGURE 6:**
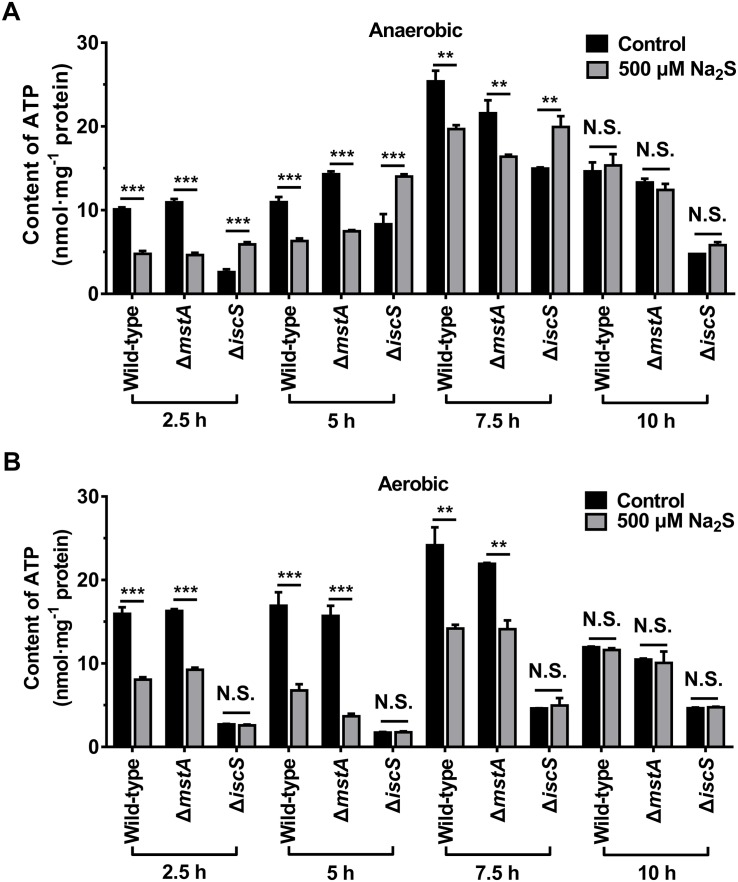
Exogenous H_2_S promotes ATP synthesis in the *E. coli* (Δ*iscS*) mutant strains in the logarithmic growth phase under anaerobic conditions. **(A)** The ATP content in cell lysates of *E. coli* under anaerobic conditions. **(B)** the ATP content in cell lysates of *E. coli* under aerobic conditions. Samples without Na_2_S treatment were used as control, and the results were normalized to the protein concentrations of the cell lysates. Each bar represents the mean ± SD of four independent experiments. ^∗∗^*p* < 0.01, ^∗∗∗^*p* < 0.001, N.S., not significant, versus the control group.

As exogenous H_2_S promoted the growth of the *E. coli* (Δ*iscS*) mutant, we hypothesized that H_2_S is involved in cellular energy metabolism by stimulating the synthesis of ATP. After a 2.5–10 h incubation of the wild-type *E. coli*, mutant *E. coli* (Δ*mstA*) and *E. coli* (Δ*iscS*) strains under anaerobic conditions, the ATP content in cell lysates was measured. As shown in Figure 6A, after incubation for 2.5 h the ATP content in the wild-type *E. coli* cells decreased from 10.1 to 4.8 nmol mg^–1^ protein after the addition of 500 μM Na_2_S to the culture, and the ATP content in the mutant *E. coli* (Δ*mstA*) cells decreased from 10.9 to 4.6 nmol mg^–1^ protein. These results suggested that 500 μM H_2_S is toxic to wild-type *E. coli* and mutant *E. coli* (Δ*mstA*) under anaerobic conditions. In contrast, the ATP content in the mutant *E. coli* (Δ*iscS*) cells increased ∼2.3-fold (from 2.6 to 5.9 nmol mg^–1^ protein.) in the presence of 500 μM Na_2_S. However, exogenous Na_2_S did not significantly affect ATP content in all three strains in the stable growth phase after incubation for 10 h (Figures 4C,D, 6A). These results showed that exogenous Na_2_S promotes ATP synthesis in the mutant *E. coli* (Δ*iscS*) during the logarithmic growth phase under anaerobic conditions.

We next measured the ATP content in the wild-type and the mutant *E. coli* cells cultured under aerobic conditions. As shown in Figure 6B, The ATP content in the wild-type and the mutant *E. coli* (Δ*mstA*) cells was greatly decreased after the addition of 500 μM Na_2_S to the culture. Exogenous Na_2_S (500–2000 μM) significantly inhibited the growth of wild-type *E. coli* under aerobic conditions (Figures 4E,G). It is, therefore, not surprising that exogenous Na_2_S inhibited the biosynthesis of ATP in the wild-type and the mutant *E. coli* (Δ*mstA*) cells. Interestingly, unlike under anaerobic conditions, the ATP content in the mutant *E. coli* (Δ*iscS*) cells cultured under aerobic conditions was not significantly affected by the addition of 500 μM Na_2_S. These results indicated that hydrogen sulfide from IscS sustains cellular bioenergetics in *E. coli* under anaerobic conditions.

Finally, we explored the mechanism of hydrogen sulfide promoting ATP synthesis in the mutant *E. coli* (Δ*iscS*) strain under anaerobic conditions. A previous study indicated that incubation with NaHS (an H_2_S donor) markedly augmented the catalytic activity of glyceraldehyde-3-phosphate dehydrogenase (GAPDH) in both purified protein and HEK293 cells (Mustafa et al., 2009). As the amino acid sequence of *E. coli* GAPDH shared 79% identity with that of human beings, we speculated that *S*-sulfhydration of GAPDH in *E. coli* would stimulate the glycolytic pathway and enhance ATP synthesis under anaerobic conditions. Recombinant *E. coli* GAPDH was purified and protein purity was judged to be >95% by SDS-PAGE analysis (Supplementary Figure S9a). Recombinant GAPDH protein was *S*-sulfhydrated when incubated with Na_2_S combined with diamide as a strong thiol-specific oxidant. Unexpectedly, unlike GAPDH from mammalian cells, incubation with Na_2_S combined with diamide did not significantly increase the *E. coli* GAPDH activity (Supplementary Figure S9b). However, a recent study indicated that GAPDH was inactivated by *S*-sulfhydration *in vitro*, which is inconsistent with the study mentioned above (Jarosz et al., 2015). Further studies will be needed to elucidate the mechanism by which exogenous H_2_S enhances ATP synthesis in the mutant *E. coli* (Δ*iscS*) under anaerobic conditions.

## Discussion

IscS is highly conserved in all living cells, from bacteria to humans, catalyzing the formation of a protein-bound persulfide and *L*-alanine using *L*-cysteine as a substrate (Lauhon et al., 2004). Obviously, a persulfide intermediate can produce either elemental sulfur or H_2_S depending on the redox state of the reaction medium. H_2_S production catalyzed by purified IscS greatly increased in the presence of DTT (Figures 3B–D). The activity of IscS was regulated under the different redox conditions, and the midpoint redox potential was determined to be −329 ± 1.6 mV. Additionally, in *E. coli* cells H_2_S production from IscS is regulated under oxidative and reductive stress (Figure 3E). Notably, the redox state of the bacterial cytoplasm is different under aerobic versus anaerobic conditions (Li et al., 2004). A recent study indicated that the intracellular redox potential undergoes reductive changes associated with the induction of hypoxia (Jiang et al., 2014). These results suggest that H_2_S generation by IscS would be regulated by the redox state of the bacterial cytoplasm under aerobic and anaerobic conditions, which is consistent with our observations in the present study.

IscS acts as a sulfur donor and is involved in the biogenesis of iron-sulfur clusters in the model bacterium *E. coli*. Iron-sulfur clusters are of great importance in the function of proteins involved in energy metabolism, including in electron transport in respiratory chain complexes, the Krebs cycle and photosynthesis (Rouault, 2012). Under oxygen-rich conditions, the Krebs cycle is a major energy-producing metabolic pathway, and a large amount of elemental sulfur generated by IscS is required for the assembly of iron-sulfur proteins. However, under oxygen-deficient conditions, we speculate that H_2_S production from IscS would promote cellular energy metabolism and ATP synthesis through an increase in substrate-level phosphorylation. Actually, exogenous Na_2_S (100–500 μM) significantly promoted the growth of the mutant *E. coli* (Δ*iscS*) in the logarithmic growth phase under anaerobic conditions, but not under aerobic conditions (Figure 4). Accordingly, the ATP content in the mutant *E. coli* (Δ*iscS*) increased ∼2.3-fold in the presence of 500 μM Na_2_S under anaerobic conditions, but was not altered under aerobic conditions (Figure 6). It should be noted, however, that elimination of iscS results in a viable organism that displays complex nutritional requirements and severely reduced activity of Fe-S cluster enzymes (Lauhon and Kambampati, 2000; Schwartz et al., 2000; Tokumoto and Takahashi, 2001; Lauhon et al., 2004). Exogenous H_2_S might promote the growth of the mutant *E. coli* (Δ*iscS*) by directly or indirectly rescuing these defects.

Additionally, in the present study it was observed that 500 μM Na_2_S is toxic to wild-type *E. coli* under both aerobic and anaerobic conditions, whereas it promotes the growth of the mutant *E. coli* (Δ*iscS*) under anaerobic conditions. The mechanism of the opposite effects of Na_2_S on the wild-type and the mutant *E. coli* (Δ*iscS*) is not known but could result from altered energy metabolism pathways. Previous studies showed that the toxicity of H_2_S has been attributed to its ability to inhibit cytochrome oxidase of the electron transport chain, resulting in the inhibition of ATP production and metabolic suppression (Cooper and Brown, 2008). In the present study, high concentrations of Na_2_S (500 μM) would inhibit cytochrome oxidase of wild-type *E. coli*, and consequently inhibited cell growth and ATP production (Korshunov et al., 2016). In the case of the mutant *E. coli* (Δ*iscS*), however, the function of electron transport in respiratory chain complexes was severely impaired due to defects in iron-sulfur cluster synthesis. Therefore, dysfunctional cytochrome oxidase or respiratory chain complexes may be insensitive to H_2_S treatment, resulting in enhanced H_2_S tolerance in the mutant *E. coli* (Δ*iscS*).

Previous studies demonstrated that elevated levels of H_2_S in mammalian mitochondria stimulates the production of ATP, which is based on the mechanism by which H_2_S donates electrons to the electron transport chain through H_2_S oxidation catalyzed by sulphide:quinone oxidoreductase (SQR) (Fu et al., 2012; Módis et al., 2016). Additionally, when sulfide levels rose, *E. coli* became strictly dependent upon cytochrome *bd* oxidase for continued respiration. The sulfide resistance of cytochrome *bd* oxidase is a key trait that permits respiration in low oxygen conditions (Forte et al., 2016; Korshunov et al., 2016). However, these mechanisms do not provide a reasonable explanation for the results obtained in the present study. The first reason is because sulfide:quinone oxidoreductase or one of its orthologs is not present in *E. coli*. Second, our results clearly indicated that exogenous H_2_S do not promote ATP synthesis in the wild-type and the mutant *E. coli* (Δ*iscS*) under aerobic conditions (Figure 6). Third, the cytochrome oxidase in *E. coli* catalyzes the transfer of electrons from reduced ubiquinone to molecular oxygen. This electron transfer contributes to the energy yield of respiration. However, molecular oxygen cannot be the terminal electron acceptor of *E. coli* respiratory chain under anaerobic conditions.

In summary, our results clearly demonstrated that IscS, not 3-MST, is the primary source of H_2_S in *E. coli* under anaerobic conditions, as evidenced by assays using chemical inhibitors and *iscS*, *mstA*, *cyuA* and *sufS* knockout strains. Hydrogen sulfide from IscS sustains cell growth and bioenergetics in *E. coli* under anaerobic conditions.

## Data Availability Statement

All datasets generated/analyzed for this study are included in the manuscript/Supplementary Files.

## Author Contributions

WN conceived and designed the experiments. JW, XG, HL, HQ, JQ, SY, and WN performed the experiments. WN, JW, and JS analyzed the data. JW and WN wrote the manuscript.

## Conflict of Interest

The authors declare that the research was conducted in the absence of any commercial or financial relationships that could be construed as a potential conflict of interest.
