# Supplementary material for: Hydrogen Sulfide From Cysteine Desulfurase, Not 3-Mercaptopyruvate Sulfurtransferase, Contributes to Sustaining Cell Growth and Bioenergetics in E. coli Under Anaerobic Conditions
Source: Front Microbiol. 2019 Oct 11;10:2357. doi: 10.3389/fmicb.2019.02357 (PMC6797615; doi:10.3389/fmicb.2019.02357)
Supplement: Supplementary file 1 [file Data_Sheet_1.docx]

Supplementary Materials

# Methods

**1.1 Glyceraldehyde-3-phosphate dehydrogenase (GAPDH) activity assay**

According to the method described by Modun and Williams (Modun and Williams 1999), the enzyme activity of purified GAPDH protein was measured by monitoring the change in absorbance at 340 nm at 37 °C in a BioTek Synergy2 Microplate Reader (Winooski, VT, USA) based on the conversion of NAD^+^ to NADH. In a total volume of 200 μl was included 40 mM triethanolamine, 50 mM Na_2_HPO_4_, 10 mM NAD^+^, 5 mM EDTA and 0.5 μg purified GAPDH protein, pH 8.6. The reactions were initiated by adding 5 mM DL-glyceraldehyde-3-phosphate. To determine the effect of Na_2_S, DTT and diamide on GAPDH activity, 1 mM Na_2_S, 1 mM DTT and 1 mM diamide were used to treat the purified GAPDH protein, respectively.

**1.2 Detection of cysteine content in *E. coli* cells**

The cysteine content in *E. coli* cells was determined using the colorimetric method with a Cysteine Assay Kit (Solarbio, Beijing, China) following the manufacturer’s instructions. The wild-type and mutant *E. coli* (Δ*isc*S) strains were cultured at 37 °C under anaerobic conditions. To determine the effect of Na_2_S on the synthesis of cysteine in *E. coli* cells, Na_2_S solution was injected into the sealed culture bottle at a final concentration of 500 μM. After an incubation of 2.5 h, the cultures were centrifuged at 4,000 rpm at 4 °C for 10 min to collect the precipitate, which was followed by washing 3 times with ice-cold PBS. The precipitate was resuspended in 500 μl of extracting solution. The cell suspension was lysed via cyclic liquid nitrogen freezing-thawing treatment processes 5 times. Subsequently, the supernatant was collected by centrifugation at 15,000 rpm at 4 °C for 5 min, and 20 μl of the lysate was added to the reaction solution. After an incubation of 15 min at 37 °C, the absorbance at 600 nm was measured by microplate reader, and the results are presented as nmol cysteine/mg protein.

**References**

Modun B., Williams P. (1999) The staphylococcal transferrin-binding protein is a cell wall glyceraldehyde-3-phosphate dehydrogenase. Infect Immun 67, 1086-1092.

| **Table S1 Primers used in this study** | | |
| --- | --- | --- |
| Primer name | Primer sequence | Use |
| *iscS-*F: | CGCGGGATCCATGAAATTACCGATTTATCTCG | Cloning of *iscS* gene |
| *iscS-*R: | CGCGCTCGAGTTAATGATGAGCCCATTCGATG | Cloning of *iscS* gene |
| *gapA-*F: | GCATGGATCCATGACTATCAAAGTAGGTATC | Cloning of *gapA* gene |
| *gapA-*R: | GCATCTCGAGTTATTTGGAGATGTGAGCGATC | Cloning of *gapA* gene |
| *mstA*-upF: | AGCACCTTACCGGCACTCTG | Amplification of *mstA* homogenous arm |
| *mstA*-upR: | CACTGGCTCAACCGGTAAATACCATGTCGTGGACATAGGC | Amplification of *mstA* homogenous arm |
| *mstA*-dnF: | ATTTACCGGTTGAGCCAGTG | Amplification of *mstA* homogenous arm |
| *mstA*-dnR: | CGAGTATTGTCTCCGCTGAG | Amplification of *mstA* homogenous arm |
| *mstA*-VeriF: | ACATCCGACAATTTAGCCT | Verification of *mstA* deletion |
| *mstA*-VeriR: | AACCGCCAAATTCGGCGGTT | Verification of *mstA* deletion |
| sg*mstA*-F: | GGAGGATCGTAACGTTGCTCGTTTTAGAGCTAGAAATAGCAAG | Amplification of sgRNA-*mstA* |
| sg*mstA*-R: | ACTAGTATTATACCTAGGAC | Amplification of sgRNA-*mstA* |
| *cyuA*-VeriF： | GATGCCTGCCGAGAAGATT | Verification of *cyuA* deletion |
| *cyuA*-VeriR： | CAGGGAGATGATGATGAGT | Verification of *cyuA* deletion |
| sg*sufS*-F: | GCGACTTTTACGTGTTCTCCGTTTTAGAGCTAGAAATAGC | Amplification of sgRNA-*sufS* |
| sg*sufS*-R: | GGAGAACACGTAAAAGTCGCACTAGTATTATACCTAGGAC | Amplification of sgRNA-*sufS* |
| *sufS*-upF: | CCCAAGCTTGGTGAAAAACGAGGTGTGT | Amplification of *sufS* homogenous arm |
| *sufS*-upR: | GGGCGCTTAAGGTATGAAT | Amplification of *sufS* homogenous arm |
| *sufS*-dnF: | AGGCTTGGCGTTATTGCTT | Amplification of *sufS* homogenous arm |
| *sufS*-dnR: | CCGCTCGAGTCGCTTCCAGACCTTGTGA | Amplification of *sufS* homogenous arm |
| *sufS-*VeriF: | CACTGCCCATTACCGACAT | Verification of *sufS* deletion |
| *sufS-*VeriR: | CTGAAACCAAAACACTCCT | Verification of *sufS* deletion |

***
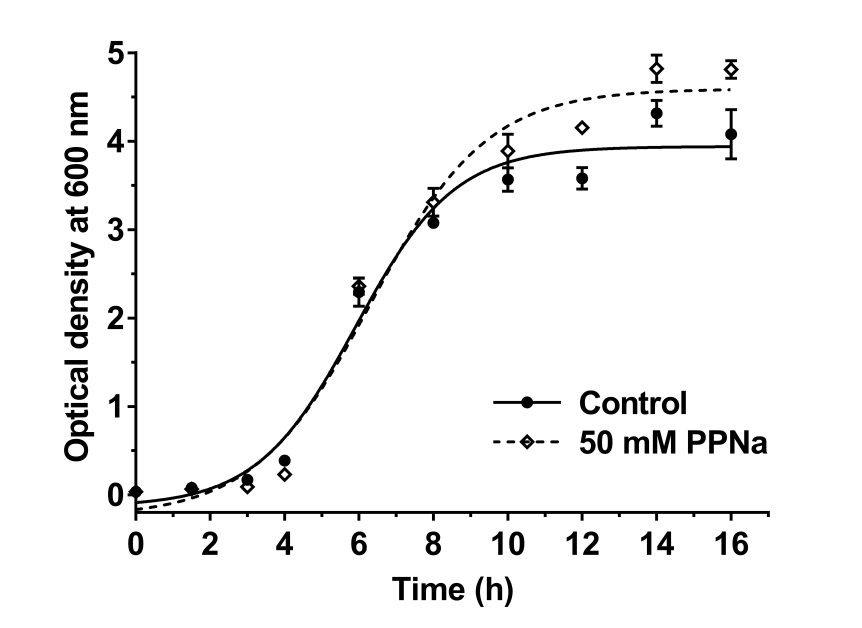
***

**Fig. S1 The growth of wild-type *E. coli* under aerobic conditions is not significantly affected by 50 mM PPNa** The group without the addition of PPNa was used as control. All the results are presented as the means ± SD (n = 4).


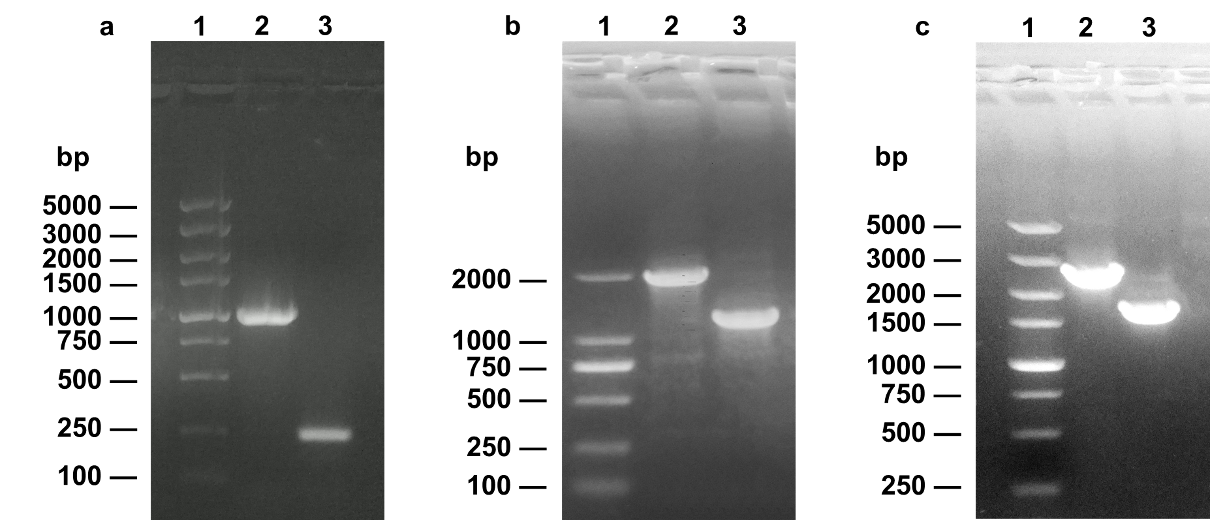


**Fig. S2 Verification of the *mstA*, *cyuA* and *sufS* deletion** The correct mutant *E. coli* (Δ*mstA*) and *E. coli* (Δ*cyuA*) strains were confirmed by agarose gel electrophoresis: **(a)** lane 1, Molecular mass standards; lane 2, wild-type *E. coli*; lane 3, *E. coli* (Δ*mstA*). The molecular weight of PCR amplicon obtained using the wild-type *E. coli* genome as template should be 1040 bp, and that obtained using the *E. coli* (Δ*mstA*) mutant genome as template should be 237 bp. **(b)** lane 1, Molecular mass standards; lane 2, wild-type *E. coli*; lane 3, *E. coli* (Δ*cyuA*). The molecular weight of PCR amplicon obtained using the wild-type *E. coli* genome as template should be 1920 bp, and that obtained using the *E. coli* (Δ*cyuA*) mutant genome as template should be 1209 bp. **(c)** lane 1, Molecular mass standards; lane 2, wild-type *E. coli*; lane 3, *E. coli* (Δ*sufS*). The molecular weight of PCR amplicon obtained using the wild-type *E. coli* genome as template should be 2505 bp, and that obtained using the *E. coli* (Δ*sufS*) mutant genome as template should be 1705 bp.


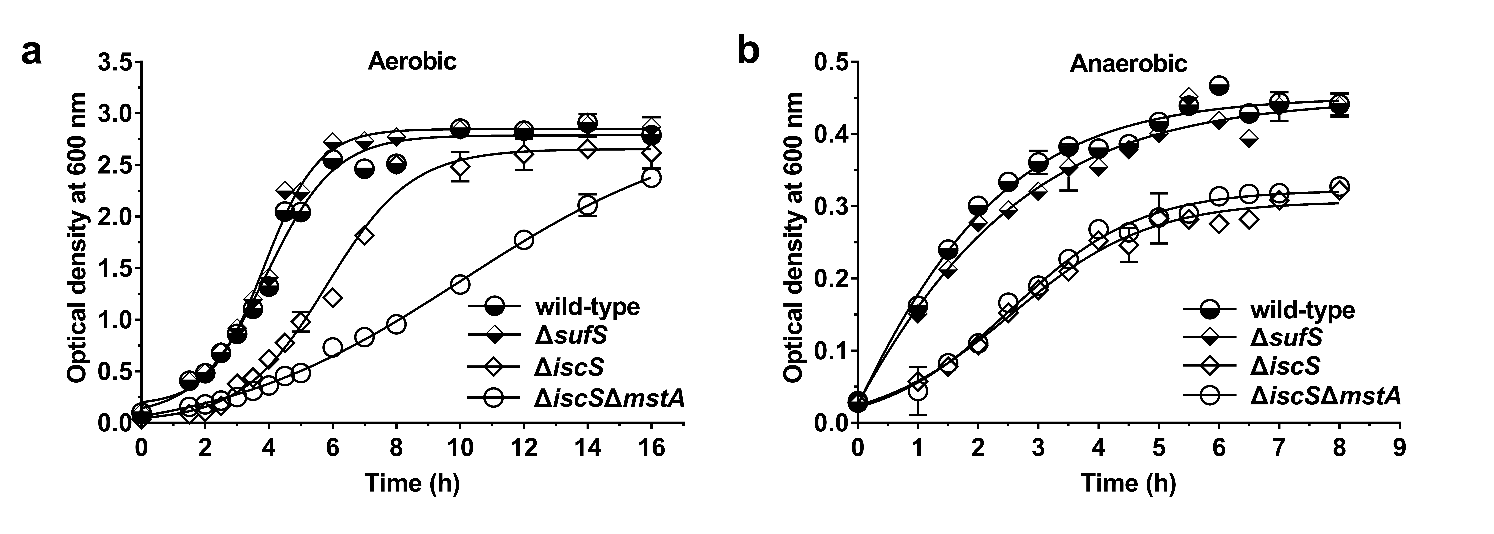


**Fig. S3 Growth curves of wild-type and mutant *E. coli* strains (a)** Growth curves of wild-type, mutant *E.coli* (Δ*sufS*), (Δ*iscS*) and (Δ*iscS*Δ*mstA*) under aerobic conditions. **(b)** Growth curves of wild-type, mutant *E.coli* (Δ*sufS*), (Δ*iscS*) and (Δ*iscS*Δ*mstA*) under anaerobic conditions. Each bar represents the mean ± SD of four independent experiments.


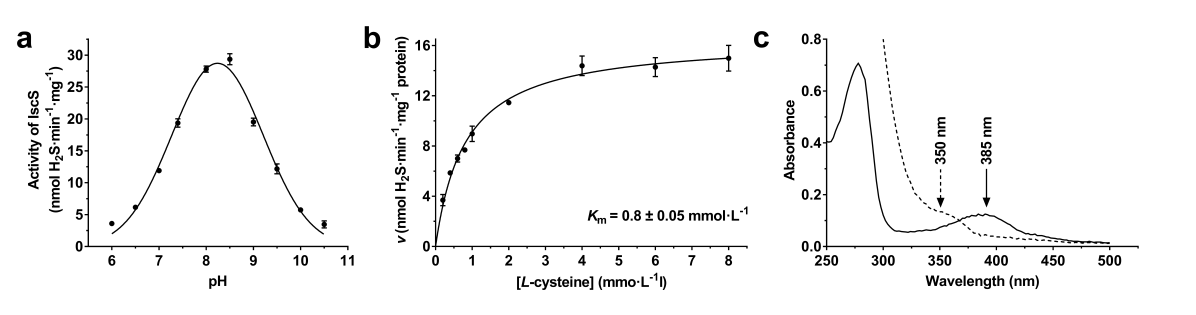


**Fig. S4 Characteristics of purified IscS protein (a)** The effect of pH on the activity of IscS was determined using two buffer systems, sodium phosphate (pH 6.0-7.5) and Tris-HCl (pH 8.0-10.5) in the presence of 1 mM DTT. **(b)** The kinetics of purified IscS activity was investigated using varying concentrations of *L*-cysteine in the presence 1 mM DTT at pH 8.0, and the kinetic constant *K*m was calculated using nonlinear regression in GraphPad Prism version 7. **(c)** Purified IscS has a characteristic absorption peak at 385 nm based on the presence of pyridoxal phosphate (PLP) as a cofactor (continuous line), and when the substrate *L*-cysteine is added, the absorption peak shifted to 350 nm due to the binding of the substrate to PLP (dotted line).


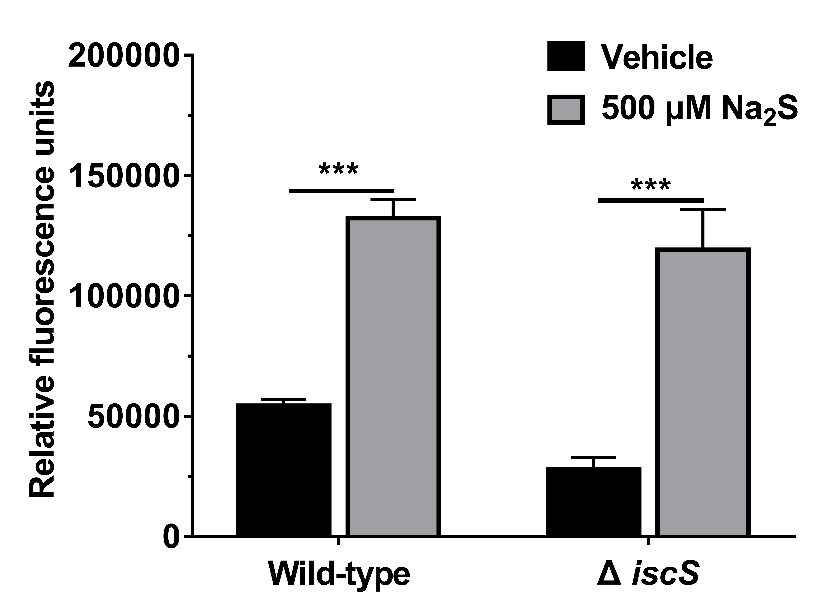


**Fig. S5 Exogenous Na_2_S significantly increased the intracellular content of H_2_S in *E. coli*** The content of H_2_S in *E. coli* cells was greatly increased by the addition of 500 μM Na_2_S into the culture medium. Relative fluorescence units were normalized to the protein content of each sample. Each bar represents the mean ± SD of four independent experiments. ****p* < 0.001.


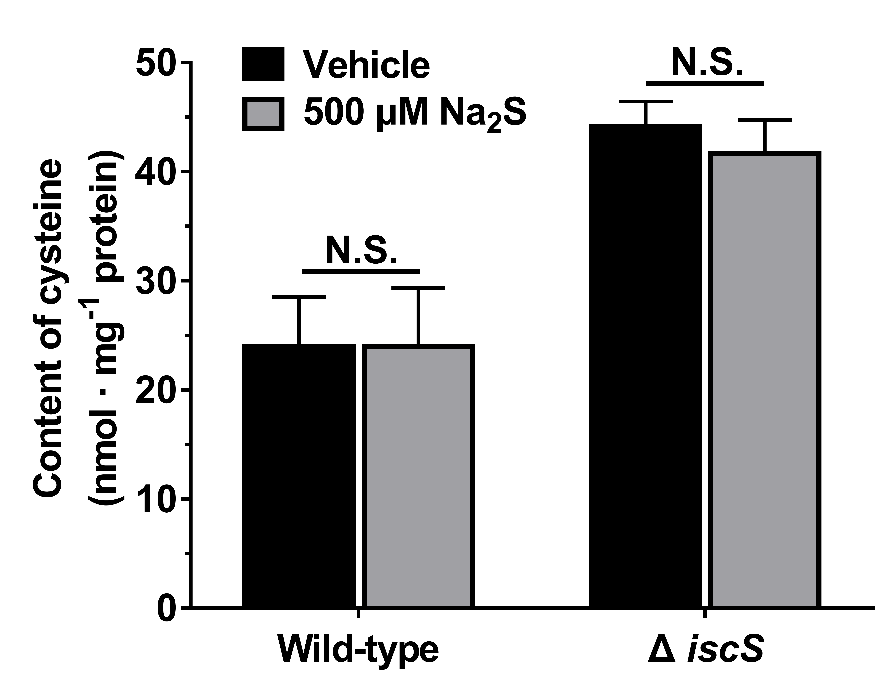


**Fig. S6 Effect of exogenous Na_2_S on the intracellular content of cysteine in *E. coli*** The cysteine content in *E. coli* cells was not significantly affected by the addition of 500 μM Na_2_S into the culture medium. The cysteine content was normalized by the protein content of the cell lysate. Each bar represents the mean ± SD of four independent experiments. N.S., not significant.


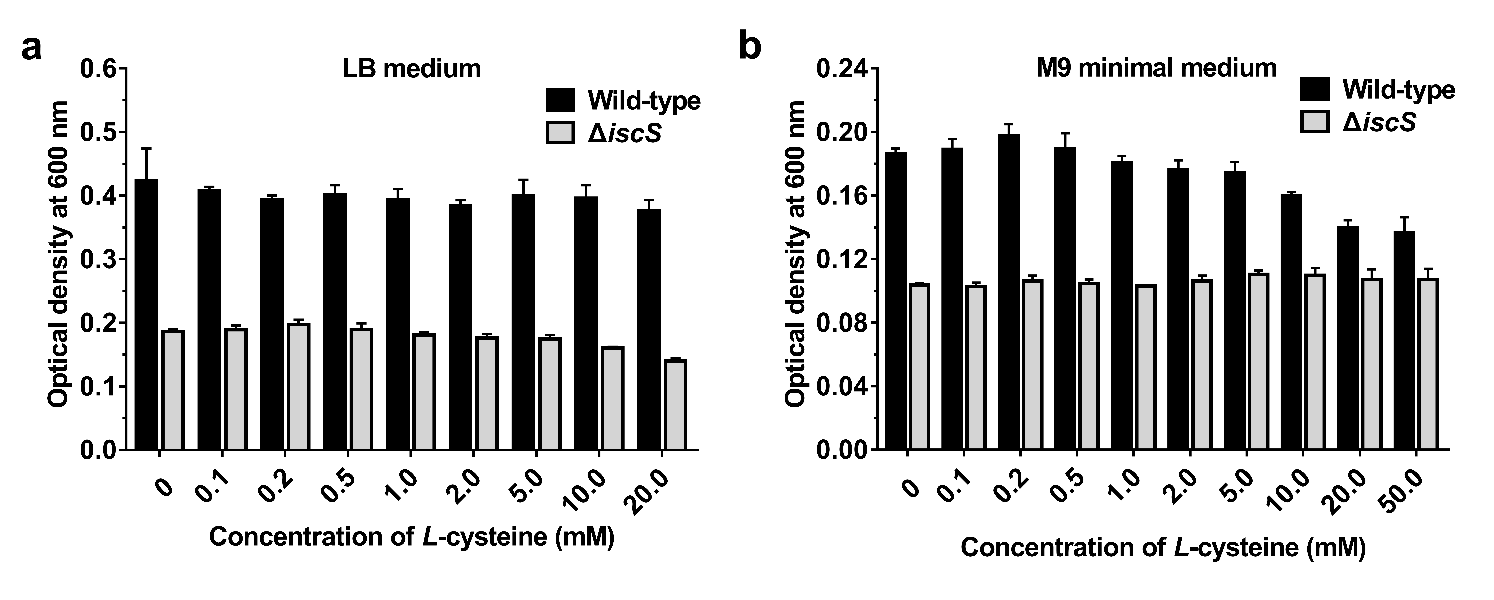


**Fig. S7 Effect of different concentrations of *L*-cysteine on the growth of *E. coli* under anaerobic conditions in LB medium and M9 minimal medium (a)** Effect of different concentrations of *L*-cysteine on the growth of wild-type *E. coli* and the *E. coli* (Δ*iscS*) mutant in LB medium under anaerobic conditions. **(b)** Effect of different concentrations of *L*-cysteine on the growth of wild-type *E. coli* and the *E. coli* (Δ*iscS*) mutant in M9 minimal medium (containing 0.4% glucose) under anaerobic conditions***.*** Fifty microliters of overnight culture (1%, v/v) was inoculated into a culture bottle containing 5 ml of LB medium with different concentrations of *L*-cysteine. After 2.5 h of incubation the OD_600_ values were determined. The group without the addition of *L*-cysteine was used as a control group. Each bar represents the mean ± SD of four independent experiments.


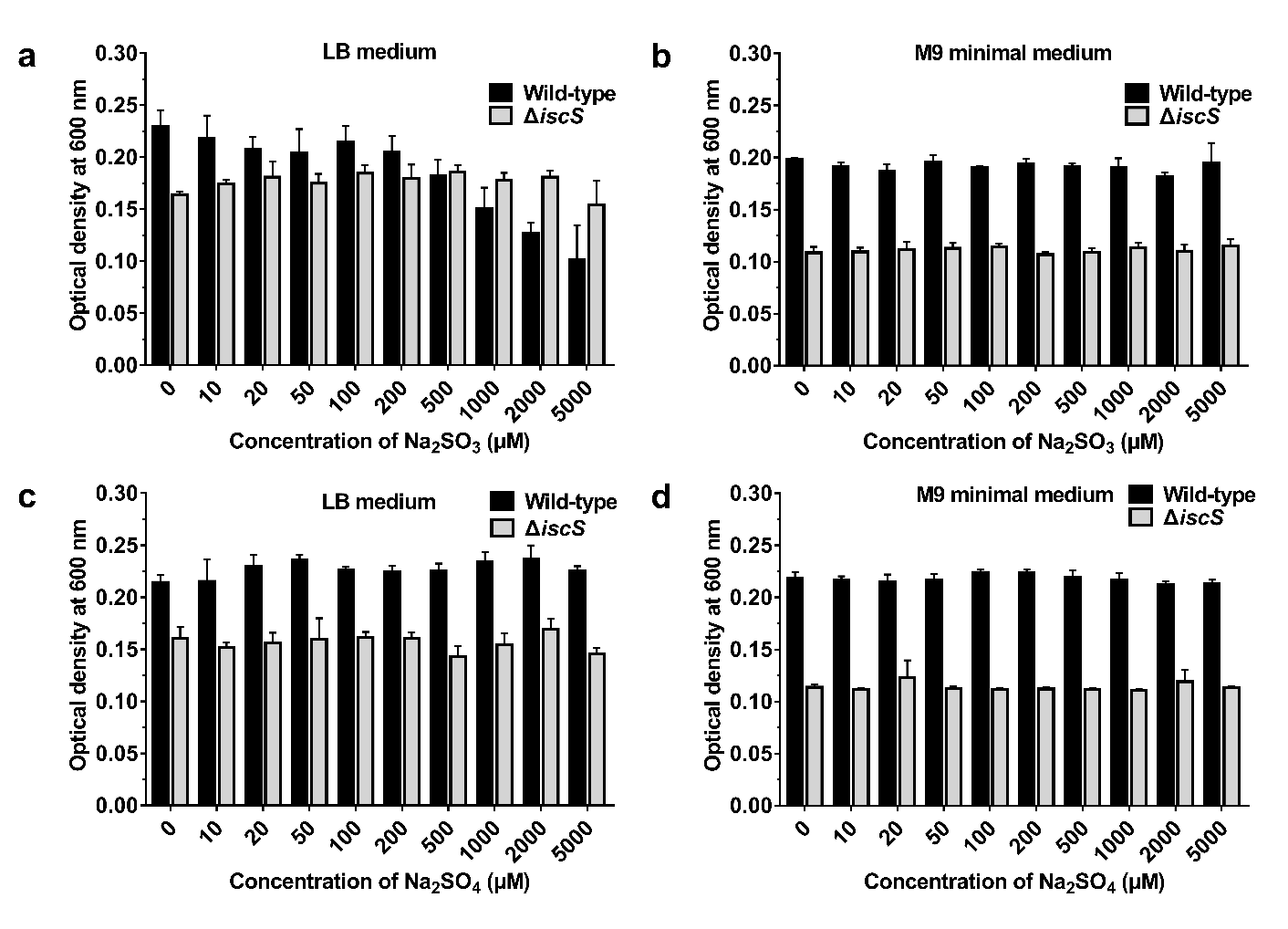


**Fig. S8 Effect of different concentrations of Na_2_SO_3_ and Na_2_SO_4_ on the growth of *E. coli* under anaerobic conditions in LB medium and M9 minimal medium (a)** Effect of different concentrations of Na_2_SO_3_ on the growth of wild-type *E. coli* and the *E. coli* (Δ*iscS*) mutant in LB medium under anaerobic conditions. **(b)** Effect of different concentrations of Na_2_SO_3_ on the growth of wild-type *E. coli* and the *E. coli* (Δ*iscS*) mutant in M9 minimal medium (containing 0.4% glucose) under anaerobic conditions. **(c)** Effect of different concentrations of Na_2_SO_4_ on the growth of wild-type *E. coli* and the *E. coli* (Δ*iscS*) mutant in LB medium under anaerobic conditions. **(d)** Effect of different concentrations of Na_2_SO_4_ on the growth of wild-type *E. coli* and the *E. coli* (Δ*iscS*) mutant in M9 minimal medium (containing 0.4% glucose) under anaerobic conditions. The group without the addition of Na_2_SO_3_ and Na_2_SO_4_ was used as a control group. Each bar represents the mean ± SD of four independent experiments.


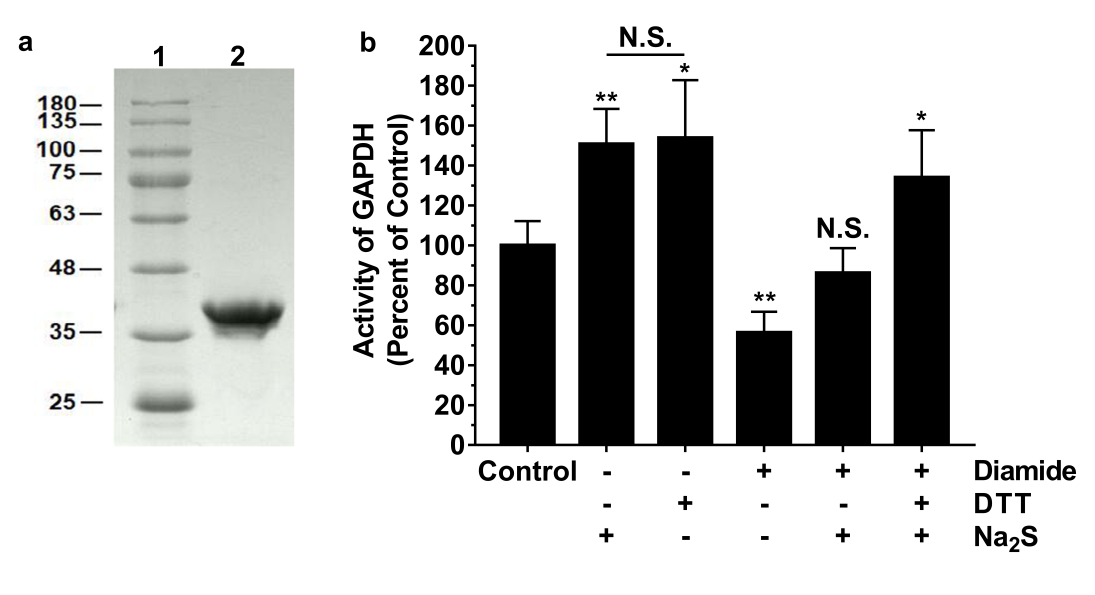


**Fig. S9** **Treatment with Na_2_S did not significantly promote GAPDH activity (a)** SDS-PAGE (12%) analysis of the purified GAPDH protein: Lane 1 - Molecular mass standards; Lane 2 - Purified GAPDH protein. **(b)** treatment with Na_2_S combined with diamide did not significantly increase the GAPDH activity. The activity of the control group was 20 umol·min^-1^·mg protein^-1^. Each bar represents the mean ± SD of four independent experiments. * *p* < 0.1, ***p* < 0.01, N.S. (not significant), versus the control group.
